# Primary mass casualty incident triage: evidence for the benefit of yearly brief re-training from a simulation study

**DOI:** 10.1186/s13049-018-0501-6

**Published:** 2018-04-27

**Authors:** Michael S. Dittmar, Philipp Wolf, Marc Bigalke, Bernhard M. Graf, Torsten Birkholz

**Affiliations:** 1 Department of Anesthesiology, Regensburg University Medical Center, Franz-Josef-Strauss-Allee 11, 93053 Regensburg, Germany; 2 Klinikum St. Marien Amberg, Emergency Department, Mariahilfbergweg 7, 92224 Amberg, Germany; 3 Department of Anesthesiology, University of Erlangen Medical Center, Krankenhausstraße 12, 91054 Erlangen, Germany

**Keywords:** Triage, Mass casualty incidents, Emergency medical services, Simulation, Training

## Abstract

**Background:**

Triage is a mainstay of early mass casualty incident (MCI) management. Standardized triage protocols aim at providing valid and reproducible results and, thus, improve triage quality. To date, there is little data supporting the extent and content of training and re-training on using such triage protocols within the Emergency Medical Services (EMS). The study objective was to assess the decline in triage skills indicating a minimum time interval for re-training. In addition, the effect of a one-hour repeating lesson on triage quality was analyzed.

**Methods:**

A dummy based trial on primary MCI triage with yearly follow-up after initial training using the ASAV algorithm (Amberg-Schwandorf Algorithm for Primary Triage) was undertaken. Triage was assessed concerning accuracy, sensitivity, specificity, over-triage, under-triage, time requirement, and a comprehensive performance measure. A subgroup analysis of professional paramedics was made.

**Results:**

Nine hundred ninety triage procedures performed by 51 providers were analyzed. At 1 year after initial training, triage accuracy and overall performance dropped significantly. Professional paramedic’s rate of correctly assigned triage categories deteriorated from 84 to 71%, and the overall performance score decreased from 95 to 90 points (maximum = 100). The observed decline in triage performance at 1 year after education made it necessary to conduct re-training. A brief didactic lecture of 45 min duration increased accuracy to 88% and the overall performance measure to 97.

**Conclusions:**

To improve disaster preparedness, triage skills should be refreshed yearly by a brief re-education of all EMS providers.

## Background

Rapid triage is a mainstay of early mass casualty incident (MCI) management. For this purpose, triage protocols have been developed, which aim at both standardizing patient assessment, and enhancing triage validity and reliability. To date, there is limited evidence concerning many important aspects of MCI triage, of which the lack of an evidence based training concept is one.

While in the literature there is some information available on study associated initial triage training, [1–5] little is known concerning the question what is the minimum or optimal extent and timing of triage re-training. Since the amount of time available for the continuing education of Emergency Medical Services (EMS) personnel is limited, an educational concept supported by scientific data is warranted.

**Fig. 1:**
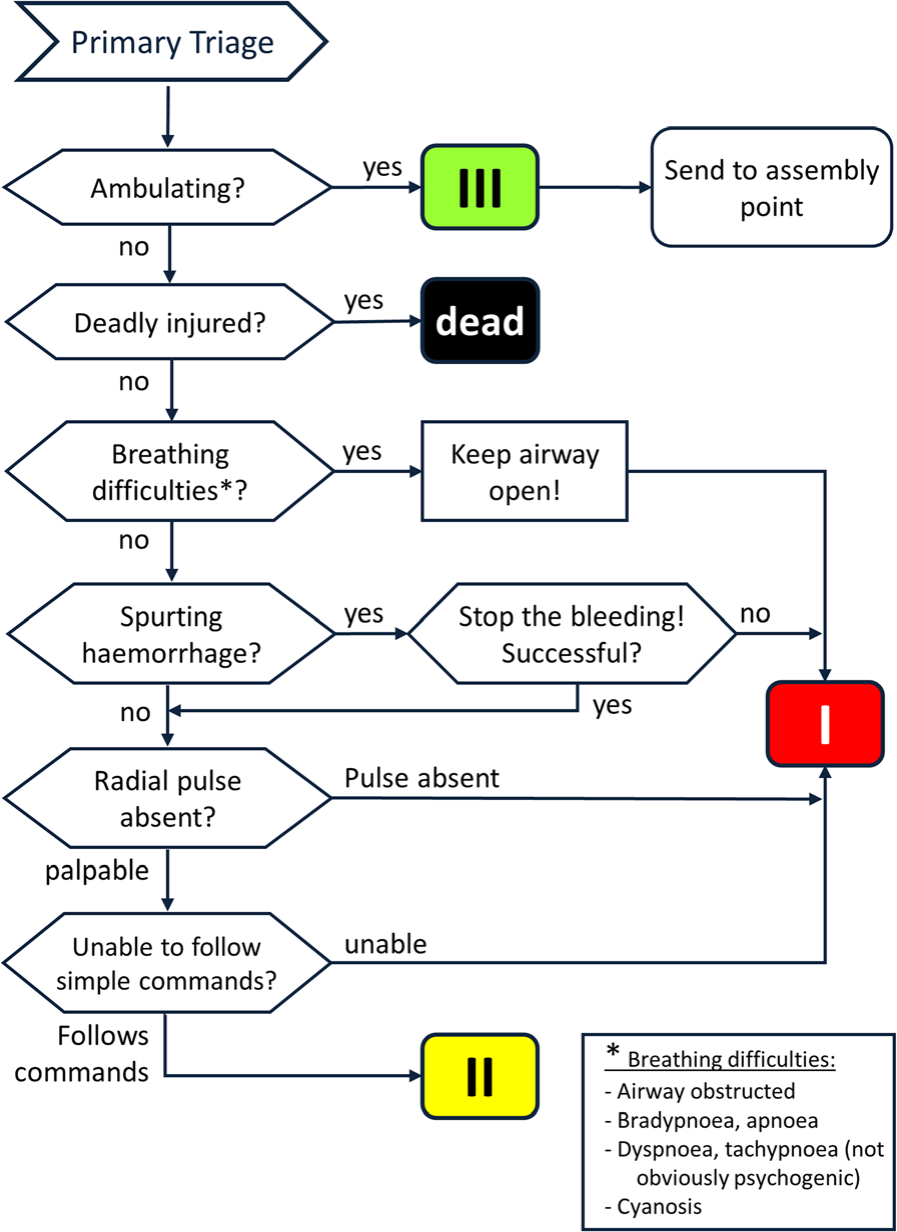
The Amberg-Schwandorf Algorithm for Primary Triage (ASAV) [4]. I = red category, II = yellow category, III = green category

In this study, the authors investigated the changes of triage performance over time subsequent to initial training, as well as the effect of a brief re-training session. The Amberg-Schwandorf Algorithm for Primary Triage (ASAV, *Amberg-Schwandorf-Algorithmus für die Vorsichtung*) (Fig. 1) served as triage algorithm for this purpose [3, 4, 6].

## Methods

The study was conducted with the approval of the local ethics committee (University of Regensburg Ethics Committee, Ref. 13–101-0001). Written informed consent was obtained from all study participants. Triage training and data acquisition was performed as reported previously in detail [3, 4].

Members of the professional EMS staff of the district of Schwandorf (Bavaria, Germany), as well as volunteers of a local disaster relief organization, participated in the study. Participants were recruited separately for each assessment session. Thus, some study participants missed one of the follow up assessments.

During the development and implementation of ASAV as a local triage algorithm, EMS personnel attended a 4 h initial triage course (3 h oral lessons, 1 h practical training on dummies), followed by a practical examination on patient dummies. The intention of the study was to reassess the participants triage performance yearly for up to three consecutive years. In case the pass rate of triage subjects (see endpoints section) would fall below 70%, the trial was planned to be stopped early to allow for premature re-qualification of the personnel. After the triage competency fell below the acceptable threshold, an additional assessment was held, to evaluate the effect of a 45 min didactical lecture refreshing triage knowledge on the triage performance.

For practical training, examinations, and data acquisition, patient dummies with written dummy description cards displaying the relevant vital data as well as additional information (such as body posture, obvious external injuries, patient demographics, and signs and symptoms to the organ systems/functions respiration, skin, bleeding, pulse status, consciousness and pain level) were used [4]. Out of a pool of 40 such vignettes, 20 were randomly selected for each triage session and brought into random order using a random number table.

### Triage modalities

Teams of two participants performed the triage process. The team leader examined the simulated patient and made the clinical decisions, while the second member documented the triage results and announced each triage step from the written algorithm, which was available to the participants throughout the triage process. After examining ten patients, the team members changed their roles and continued for the subsequent ten vignettes. During triage, a written version of the triage algorithm was available to the providers.

According to the triage algorithm, simulated patients were assigned to one of four triage categories (red = immediate treatment and/or transport, yellow = delayed treatment and transport, green = minor injuries, and black = dead). The triage team tagged red, yellow, and black patients with a plastic band of the respective color. Green patients were not tagged.

When indicated, the team leader simulated the application of bleeding control measures and/or the introduction of an oropharyngeal tube by simply placing the respective material on the patient dummy. Thus, these measures did not consume a relevant amount of time.

The triage procedure was observed by two study assistants who measured the time needed for each patient triage, rated the team performance according to predefined criteria, and documented the results.

### Endpoints

For the evaluation of triage accuracy, the resulting triage category was compared to a predefined, consented standard solution. For details on the consensus process refer to Wolf et al. [4]. Discrepancies were categorized according to Table 1. Subsequently, rates of correct triage as well as those of (critical) under- and over-triage were calculated. Furthermore, sensitivity and specificity for red patients were calculated. In an analogous manner, the accuracy of decisions concerning bleeding control and airway maneuvers was reported.

**Table 1:** Error Table

		Expected Triage Category			
		Red	Yellow	Green	Dead
Triaged as	Red	Ok	Critical Over	Critical Over	Critical Over
	Yellow	Critical Under	Ok	Over	Over
	Green	Critical Under	Under	Ok	Over
	Dead	Critical Under	Under	Under	Ok

Classification of triage errors according to [1, 2]

To assess overall triage performance of the providers, a triage performance score was introduced. It consists of ten items (Table 2), which were rated for each individual triage procedure as being fulfilled (one point) or not (no point), and summed up to result in the final triage performance measure. Team leader and triage assistant were rated separately. The maximum score that could be gathered in 20 triage procedures was 100 points for each team member. If a score of 90 or more was achieved, the respective EMS provider was deemed qualified for performing triage. The fraction of subjects that reached this threshold is reported as pass rate.

**Table 2:** Triage performance measure

Team leader (maximum 6 points per patient)	Triage assistant (maximum 4 points per patient)
Correct role behavior	Correct role behavior
Explicit communication	Explicit communication
Correct indication for bleeding control	Compliance to algorithm steps
Correct indication for Airway control	Correct documentation
Correct triage category	
Correct triage labeling	

Depending on the current role of the provider, different criteria were checked. Each criterion is rated with 1 point if fulfilled correctly or no point if not

Time requirements for each individual triage procedure were taken from the arrival at one patient until the arrival at the subsequent patient. Thus, walking times between patients were included into the measurement.

### Statistics

Statistical analysis was performed using IBM® SPSS® Statistics Version 24 (IBM Corporation, Armonk, NY, United States). Values are expressed as mean and 95% confidence intervals. Since the study participant groups at the different time points differed concerning their qualification (paramedic (in Germany *Rettungsassistent*), emergency medical technician (in Germany *Rettungssanitäter*), others (mostly inferior qualification)) and employment status (professional, volunteer), professional paramedics as largest subgroup among the participants were analyzed further.

Parameters concerning triage procedures (as specified under Part A), such as sensitivity, specificity, (critical) over- and under-triage, were compared between time points by one-way analysis of variances (ANOVA) with Bonferroni post-hoc testing.

Since triage duration is depending on the triage category [4], and since for didactical reasons red and yellow patients were over-represented in the patient collective, time requirement data were weighed to represent a realistic distribution of resulting triage categories for each assessed time point. A patient distribution of 20% red, 20% yellow, and 60% green patients was assumed [7].

Provider based parameters (as specified under Part B) were compared using paired statistical tests. Overall triage performance was compared by the paired T test, and the examination pass rate (> = 90% triage performance) by the non-parametric paired Wilcoxon test. Triage performance data were analyzed for normal distribution by the Shapiro-Wilk-Test.

*P* values below 0.05 were considered statistically significant.

## Results

Since triage performance had decreased substantially after 1 year [3], the observational part of the study was stopped before the two-year follow-up data acquisition to allow a prior re-training.

The study participant characteristics concerning qualification and employment status are displayed in Table 3.

**Table 3:** Participant characteristics (Part B)

	Initial assessment	2nd assessment	3rd assessment (after re-training)
Qualification			
Paramedic (*Rettungsassistent*)	19 (57.6%)	10 (45.5%)	14 (73.7%)
EMT (*Rettungssanitäter*)	4 (12.1%)	3 (13.6%)	4 (21.1%)
Other	10 (30.3%)	9 (40.9%)	1 (5.3%)
Total	33 (100%)	22 (100%)	19 (100%)
Employment			
Professional	24 (72.7%)	13 (59.1%)	19 (100.0%)
Volunteer	9 (27.3%)	9 (40.9%)	0 (0.0%)
Total	33 (100%)	22 (100%)	19 (100%)

### Part A: analysis of triage procedures

**Fig. 2:**
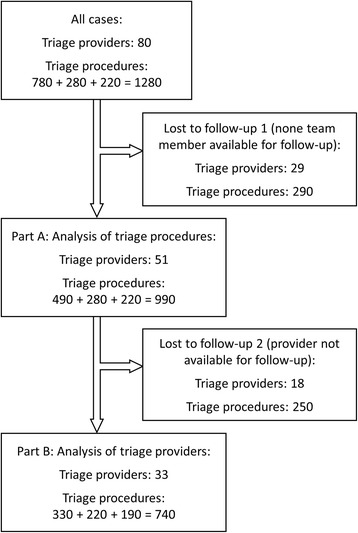
Case processing. N of triage procedures is displayed as triage runs at baseline + runs at 1 year + runs at 2 years / after re-training

During the course of examination, the study assistants evaluated 80 triage providers performing 1280 triage procedures (780 during initial training, 280 subsequent to 1 year, and 220 after re-training at around 2 years). For analysis, triage runs were removed, if none of the two team members participated in the baseline evaluation and at least one of the follow-up sessions. Thus, N1 = 990 triage procedures (490 + 280 + 220) performed by 51 providers were included into the analysis of triage procedures (Part A) (Fig. 2).

Follow-ups were undertaken at a mean latency of 14.6 and 10.5 months, respectively. After 1 year, under-triage was more frequent, as demonstrated by specificity being higher than sensitivity. At this time point, the triage quality had significantly decreased in respect to the rate of under-triage, critical under-triage, over-triage, critical over-triage, as well as the accuracy of airway handling measures and bleeding control measures (Table 4). Compared to the one-year follow-up, the brief re-training session led to a significant improvement concerning sensitivity, under-triage, critical under-triage, airway and bleeding management accuracy, and time requirement. The accuracy of airway measures was even superior to the results reached by initial training (Table 4).

**Table 4:** Overview over the study results for all participants

Part A: Level of triage procedures	All (N1 = 990)			Statistical comparisons			Test
	Initial assessment (N1 = 490)	2nd assessment (N1 = 280)	3rd assessment (after re-training) (N1 = 220)	2nd vs. Initial	3rd vs. Initial	3rd vs. 2nd	
Time since initial training (months)	0	14.6 (14.0–15.3)	25.1 (22.9–27.3)				
Accuracy (triage category correct)	84% (80–87)	77% (69–85)	86% (82–91)	0.159	1.000	0.069	A
Sensitivity	86% (82–91)	77% (69–85)	92% (86–98)	0.058	0.707	**0.012 ⇑**	A
Specificity	91% (87–94)	85% (79–90)	89% (84–94)	0.182	1.000	0.767	A
Under-triage	10% (8–13)	19% (12–27)	5% (2–8)	**0.012 ⇓**	0.360	**0.000 ⇑**	A
Critical under-triage	6% (4–8)	13% (6–20)	3% (1–5)	**0.026 ⇓**	1.000	**0.007 ⇑**	A
Over-triage	6% (4–8)	13% (6–20)	9% (5–13)	**0.049 ⇓**	1.000	0.726	A
Critical over-triage	4% (3–6)	12% (5–19)	7% (4–11)	**0.022 ⇓**	0.982	0.535	A
Airway handling accurate	91% (88–93)	81% (76–86)	97% (95–99)	**0.000 ⇓**	**0.025 ⇑**	**0.000 ⇑**	A
Bleeding management accurate	93% (91–96)	84% (80–89)	94% (90–97)	**0.000 ⇓**	1.000	**0.001 ⇑**	A
Time requirement (sec)	35.4 (33.9–37.0)	36.9 (34.2–39.6)	28.2 (25.8–30.6)	0.917	**0.000 ⇑**	**0.000 ⇑**	A
Time requirement (sec) weighed	25.0 (23.4–26.6)	27.4 (24.7–30.1)	22.2 (19.9–24.6)	0.305	0.228	**0.009 ⇑**	A
Part B: Level of providers	All			Statistical comparisons			Test
	Initial assessment (N2 = 33)	2nd assessment (N2 = 22)	3rd assessment (after re-training) (N2 = 19)	2nd vs. Initial	3rd vs. Initial	3rd vs. 2nd	
Performance measure	95 (94–96)	91 (88–93)	96 (95–98)	**0.000 ⇓**	0.336	**0.003 ⇑**	T
Pass rate (≥ 90% performance)	91% (82–100)	50% (27–73)	89% (74–100)	**0.002 ⇓**	0.655	**0.014 ⇑**	W

Values are means and 95% confidence intervals. *N1* number of triage procedures analyzed, *N2* number of triage providers analyzed. Statistical testing: *A* ANOVA, *T* paired T-Test, *W* Wilcoxon Test. Bold text indicates statistically significant changes. **⇓** indicates decline; **⇑** indicates improvement

At the one-year follow-up, triage procedures performed by professional paramedics had a significantly lower accuracy in assigning the triage category, as well as airway and bleeding handling measures. Re-training significantly improved triage categorization accuracy and airway and bleeding management, as well as sensitivity, under-triage and critical under-triage (Table 5).

**Table 5:** Overview over the study results for professional paramedics only

Part A: Level of triage procedures	Professional Paramedics only (N1 = 460)			Statistical comparisons			Test
	Initial assessment (N1 = 210)	2nd assessment (N1 = 90)	3rd assessment (after re-training) (N1 = 160)	2nd vs. Initial	3rd vs. Initial	3rd vs. 2nd	
Time since initial training (months)	0	15.2 (15.0–15.7)	25.0 (22.0–28.2)				
Accuracy (triage category correct)	84% (79–89)	71% (62–81)	88% (82–93)	**0.024 ⇓**	1.000	**0.003 ⇑**	A
Sensitivity	88% (81–95)	73% (58–88)	93% (86–100)	0.073	1.000	**0.016 ⇑**	A
Specificity	88% (82–94)	79% (68–91)	87% (81–94)	0.355	1.000	0.496	A
Under-triage	10% (6–14)	16% (8–23)	4% (1–7)	0.370	0.104	**0.005 ⇑**	A
Critical under-triage	5% (2–8)	11% (4–18)	3% (0–5)	0.121	0.733	**0.012 ⇑**	A
Over-triage	6% (3–10)	13% (6–20)	9% (5–14)	0.137	0.861	0.860	A
Critical over-triage	6% (3–9)	12% (5–19)	8% (4–12)	0.169	1.000	0.743	A
Airway handling accurate	91% (87–95)	77% (68–86)	98% (95–100)	**0.000 ⇓**	0.088	**0.000 ⇑**	A
Bleeding management accurate	93% (89–96)	83% (75–91)	94% (91–98)	**0.019 ⇓**	1.000	**0.008 ⇑**	A
Time requirement (sec)	34.1 (31.8–36.4)	29.1 (25.8–32.3)	29.5 (26.6–32.3)	0.063	**0.031 ⇓**	1.000	A
Time requirement (sec) weighed	23.7 (21.2–26.1)	20.7 (17.5–23.9)	23.2 (20.4–25.9)	0.513	1.000	0.815	A
Part B: Level of providers	Professional Paramedics only			Statistical comparisons			Test
	Initial assessment (N2 = 18)	2nd assessment (N2 = 9)	3rd assessment (after re-training) (N2 = 14)	2nd vs. Initial	3rd vs. Initial	3rd vs. 2nd	
Performance measure	95 (93–96)	90 (87–92)	97 (95–98)	**0.023 ⇓**	0.209	**0.061 ⇑**	T
Pass rate (≥ 90% performance)	89% (72–100)	33% (0–69)	93% (76–100)	**0.014 ⇓**	0.564	**0.046 ⇑**	W

Values are means and 95% confidence intervals. *N1* number of triage procedures analyzed, *N2* number of triage providers analyzed. Statistical testing: *A* ANOVA, *T* paired T-Test, *W* Wilcoxon Test. Bold text indicates statistically significant changes. **⇓** indicates decline; **⇑** indicates improvement

### Part B: analysis of triage providers

In this analysis, all triage providers were included, which participated in the baseline and in one or more of the follow-up sessions (N2 = 33). These providers performed 740 triage procedures (330 + 220 + 190). Triage performance data was approximately normally distributed, as assessed by the Shapiro-Wilk-Test, except for the performance score of professional paramedics at 1 year.

**Fig. 3:**
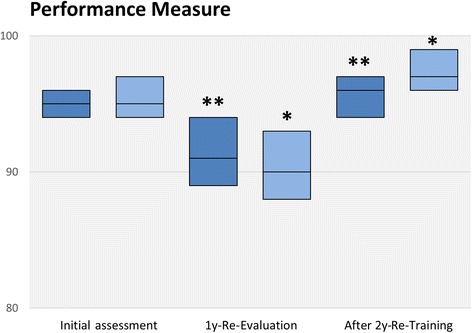
Triage Performance Results, Means and 95% confidence intervals of the triage performance measure. Dark blue: all participants, light blue: professional paramedics only. * *P* < 0.05, ** *P* < 0.01 compared to previous assessment time point, respectively

**Fig. 4:**
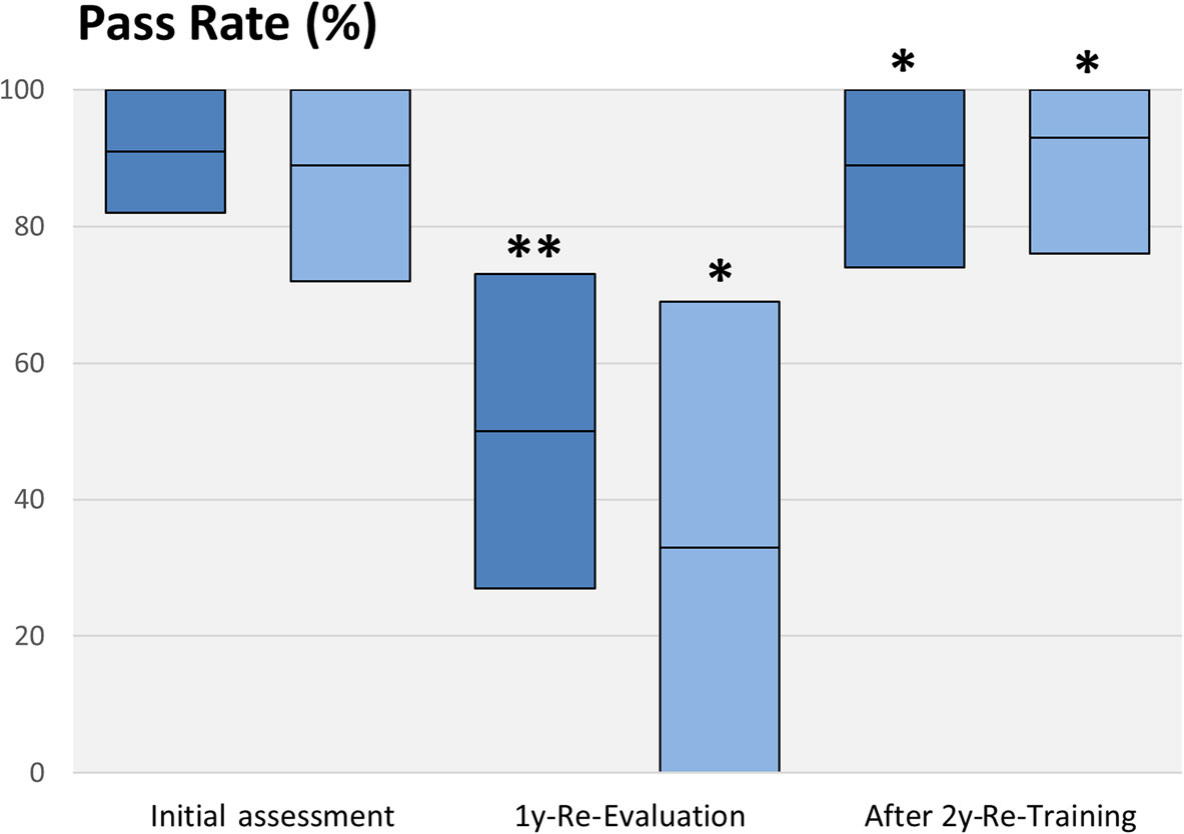
Pass Rate, Virtual pass rate at the triage examination (threshold 90 or more points at the triage performance measure). Dark blue: all participants, light blue: professional paramedics only. * *P* < 0.05, ** *P* < 0.01 compared to previous assessment time point, respectively

Overall triage performance, as measured by the performance measure, decreased significantly from initial training until the one-year follow-up. Brief re-training restored the initial performance level (Table 4, Fig. 3). For the certification pass rate (performance score of 90 or more), similar observations were made. The pass rate dropped significantly from 91 to 50%, and increased subsequent to re-training to 89% (Table 4, Fig. 4).

For the subgroup of professional paramedics, triage performance decreased significantly from 95 to 90 points, but improved after re-training to 97 points. The pass rate dropped markedly to 33% at 1 year, and increased after attending re-training to 93% (Table 5).

## Discussion

Since the development of the Simple Triage and Rapid Treatment (START) triage protocol in 1983 [8], numerous such approaches have been published in the literature. However, there is no evidence of one being clearly superior over the others [9–15]. In addition, to date there is no evidence based concept for education and re-training of these algorithms available.

Triage education typically consists of theoretical lessons followed by practical training, [1, 2, 4] or lessons without further practical consolidation [16, 17]. In some concepts, practical education is realized by computer based simulation [2] or interactive oral case discussions [1]. One study reported video based teaching combined with written and moulage training scenarios [5]. The authorities of the State of Bavaria mandate for an initial training session of 3 h, and a yearly repetition of 45 min duration [18].

Concerning the need for re-training, there are two studies that show a rapid deterioration of triage skills after initial education. Risavi and colleagues found a significant reduction in paper based and moulage triage performance at 6 months [5]. In an evaluation of the Sort, Assess, Life-saving Interventions, Treatment/Transport (SALT) algorithm, triage capabilities were reduced as early as 3 months after training [1]. There is no data available evaluating the effect of brief re-education for primary MCI triage.

In this study, data demonstrate that after the course of 1 year after initial training, the skill level has deteriorated to a degree, which is not sufficient for providing high quality triage. The threshold for triggering premature re-training was set at a pass rate of below 70%. At this level, the calculated probability that at least one member of any given ambulance team still met the qualification criteria was around 90%. Because of the loss of triage skills, the observational part of this study has been terminated to allow re-training instead of further surveying triage ability evolution. A brief oral recapitulation of the matter was sufficient to restore practical triage skills in this study.

### Triage algorithm specific results

ASAV algorithm is a functional derivative of the modified Simple Triage and Rapid Treatment (mSTART) algorithm and belongs to the START-algorithm family [6]. Both the mSTART and ASAV algorithm are prone to under-triage regarding severe brain trauma and intoxication [2, 4, 17]. As expected, the total proportion of under-triage was generally higher in all groups. Fortunately, significantly less under-triage was observed after re-training. In contrast, results for – *quoad vitam* less critical – over-triage did not change significantly after re-training. As this finding is not readily explained, one might assume that the psychological barrier to commit an over-triage is lower.

### Time requirements

The time required to finish an individual triage procedure is depending on the triage category: red and yellow patients require significantly more time than green and dead ones [4]. Since the patient vignette assignment for different assessment sessions was made by random, the distribution of triage categories differed. In addition, red and yellow patients were overrepresented in the patient collective for didactical reasons. To make the time intervals comparable between groups and to come closer to real world incidents, time measurements for the different triage categories were weighed to build an idealized patient cohort. Patient cohort distribution followed the assumptions recommended by the German consensus conference on MCI triage in 2012 (20% red and yellow respectively, 60% green) [7]. Over all participants, weighed time requirements showed a positive development after re-training. However, there was no significant change in the professional paramedic group. As the professional paramedic group’s total time consumption per triage procedure was at the lower margin of the dataset, there may have limited potential for further acceleration after re-training.

### Limitations

There are no insights into time dependent effects beyond observed intervals. Especially, the duration of the re-training’s effect could not be determined. The authors speculate, that a revision of triage associated learning matter is necessary at least every twelve months. Whether a recapitulation of MCI knowledge can be successfully accomplished by a computer based learning session to save resources remains unknown, but could serve as a further subject of research.

The current study was undertaken on patient dummies and in an artificial setting. Therefore, on the one hand, the significance of the results for real life scenarios remains uncertain. On the other hand, performing prospective research on actual mass-casualty incidents is challenging, or even impossible. Thus, MCI research to date is mostly based on case reviews, registry data analysis, and experimental research as presented here.

Since individual participants where tested repeatedly at the identical setup, some of the observed improvement in triage performance might be explained by a re-test effect. Due to the fact that the study participants were trained on the triage simulation already before the initial assessment, the long latency of around 1 year between the evaluation runs, and the marked decrease in triage performance at the second assessment make it unlikely that a re-test effect contributes significantly to the improvement seen at after re-training.

There was a significant loss-to-follow-up in study participants, which changed the composition of the participant group in respect to qualification and employment status. For the second follow-up, no volunteer personnel could be recruited. To address a potential selection bias, a subgroup analysis of professional paramedics was made, which confirmed both the deterioration of triage performance after 1 year, and the benefit of re-training.

## Conclusions

Primary triage represents an important component of MCI management. However, many aspects thereof still lack scientific foundation. It is demonstrated, that triage skills deteriorate significantly and relevantly within the first year after initial training. A brief, 45-min re-training session is capable of restoring the practical triage capabilities of professional EMS personnel. Thus, triage education should be refreshed on a yearly basis.

## References

[1] CWC L, SL ML, Peddle MB (2015). First responder accuracy using SALT after brief initial training. Prehosp Disaster Med.

[2] Gutsch W, Huppertz T, Zollner C, Hornburger P, Kay MV, Kreimeier U, Schäuble W, Kanz KG (2006). Initiale Sichtung durch Rettungsassistenten. Notfall Rettungsmed.

[3] Dittmar MS, Wolf P, Bigalke M, Graf BM, Birkholz T (2016). Nichtärztliche Vorsichtung beim Massenanfall von Verletzten: Handlungskompetenz lässt innerhalb eines Jahres deutlich nach. Notfall Rettungsmed.

[4] Wolf P, Bigalke M, Graf BM, Birkholz T, Dittmar MS (2014). Evaluation of a novel algorithm for primary mass casualty triage by paramedics in a physician manned EMS system: a dummy based trial. Scand J Trauma Resusc Emerg Med.

[5] Risavi BL, Terrell MA, Lee W, Holsten DL (2013). Prehospital mass-casualty triage training-written versus moulage scenarios: how much do EMS providers retain?. Prehosp Disaster Med.

[6] Dittmar MS, Bigalke M, Brunner A, Hannewald W, Honig D, Honig M, Kiener W, Kopf H, Schmidt T, Seeliger J, Birkholz T (2013). Ein regional angepasstes Vorgehen zur Vorsichtung und Sichtungskennzeichnung beim Massenanfall von Verletzten. Notarzt.

[7] Weidringer JW, Sefrin P, Grinda C, Weiss W (2013): Vierte Sichtungs-Konsensus-Konferenz der Schutzkommission beim Bundesministerium des Innern in Berlin am 29.10.2012.

[8] Benson M, Koenig KL, Schultz CH (1996). Disaster triage: START, then SAVE--a new method of dynamic triage for victims of a catastrophic earthquake. Prehosp Disaster Med.

[9] Kilner TM, Brace SJ, Cooke MW, Stallard N, Bleetman A, Perkins GD (2011). In ‘big bang’ major incidents do triage tools accurately predict clinical priority?: a systematic review of the literature. Injury.

[10] Garner A, Lee A, Harrison K, Schultz CH (2001). Comparative analysis of multiple-casualty incident triage algorithms. Ann Emerg Med.

[11] Lerner EB, Cone DC, Weinstein ES, Schwartz RB, Coule PL, Cronin M, Wedmore IS, Bulger EM, Mulligan DA, Swienton RE, Sasser SM, Shah UA, Weireter LJ, Sanddal TL, Lairet J, Markenson D, Romig L, Lord G, Salomone J, O'Connor R, Hunt RC (2011). Mass casualty triage: an evaluation of the science and refinement of a National Guideline. Disaster Med Public Health Prep.

[12] Lerner EB, Schwartz RB, Coule PL, Weinstein ES, Cone DC, Hunt RC, Sasser SM, Liu JM, Nudell NG, Wedmore IS, Hammond J, Bulger EM, Salomone JP, Sanddal TL, Markenson D, O'Connor RE (2008). Mass casualty triage: an evaluation of the data and development of a proposed national guideline. Disaster Med Public Health Prep.

[13] Streckbein S, Kohlmann T, Luxen J, Birkholz T, Pruckner S (2016). Sichtungskonzepte bei Massenanfallen von Verletzten und Erkrankten. Unfallchirurg.

[14] Chen J-H, Yang J, Yang Y, Zheng J-C (2015). Mass casualty incident primary triage methods in China. Chinese Med J-Peking.

[15] Heller AR, Salvador N, Frank M, Schiffner J, Kipke R, Kleber C (2017). Diagnostic precision of triage algorithms for mass casualty incidents. Anaesthesist.

[16] Deluhery MR, Lerner EB, Pirrallo RG, Schwartz RB (2011). Paramedic accuracy using SALT triage after a brief initial training. Prehosp Emerg Care.

[17] Paul AO, Kay MV, Huppertz T, Mair F, Dierking Y, Hornburger P, Mutschler W, Kanz K-G (2009). Validierung der Vorsichtung nach dem mSTaRT-Algorithmus. Unfallchirurg.

[18] Bavarian Ministry of the Interior, for Building and Transport (Bayerisches Staatsministerium des Innern, für Bau und Verkehr). Richtlinie zur Bewältigung von Ereignissen mit einem Massenanfall von Notfallpatienten und Betroffenen (MAN-RL). 2016; http://www.sfsg.de/downloads/fachbereich-menschenfuehrung.html?no_cache=1&download=2016-12-06_Hinweise_MANV_Endversion.pdf&did=871.

